# Extracellular Signal-Regulated Kinases Modulate DNA Damage Response - A Contributing Factor to Using MEK Inhibitors in Cancer Therapy

**DOI:** 10.2174/092986711798194388

**Published:** 2011-12

**Authors:** F Wei, J Yan, D Tang

**Affiliations:** 1 Division of Nephrology, Department of Medicine, McMaster University; 2 Division of Urology, Department of Surgery, McMaster University; 3 Father Sean O’Sullivan Research Institute, St. Josephs Hospital, Hamilton, Ontario, Canada;; 4 the Hamilton Center for Kidney Research, St. Joseph’s Hospital, Hamilton, Ontario, Canada; 5 The Genetics Laboratory, Institute of Women and Children’s Health, Longgong District, Shenzhen, Guangdong, P.R. China

**Keywords:** ERK1/2 kinases, DNA damage response (DDR), checkpoint activation, ATM, ATR.

## Abstract

The Raf-MEK-ERK pathway is commonly activated in human cancers, largely attributable to the extracellular signal-regulated kinases (ERKs) being a common downstream target of growth factor receptors, Ras, and Raf. Elevation of these up-stream signals occurs frequently in a variety of malignancies and ERK kinases play critical roles in promoting cell proliferation. Therefore, inhibition of MEK-mediated ERK activation is very appealing in cancer therapy. Consequently, numerous MEK inhibitors have been developed over the years. However, clinical trials have yet to produce overwhelming support for using MEK inhibitors in cancer therapy. Although complex reasons may have contributed to this outcome, an alternative possibility is that the MEK-ERK pathway may not solely provide proliferation signals to malignancies, the central scientific rationale in developing MEK inhibitors for cancer therapy. Recent developments may support this alternative possibility. Accumulating evidence now demonstrated that the MEK-ERK pathway contributes to the proper execution of cellular DNA damage response (DDR), a major pathway of tumor suppression. During DDR, the MEK-ERK pathway is commonly activated, which facilitates the proper activation of DDR checkpoints to prevent cell division. Inhibition of MEK-mediated ERK activation, therefore, compromises checkpoint activation. As a result, cells may continue to proliferate in the presence of DNA lesions, leading to the accumulation of mutations and thereby promoting tumorigenesis. Alternatively, reduction in checkpoint activation may prevent efficient repair of DNA damages, which may cause apoptosis or cell catastrophe, thereby enhancing chemotherapy’s efficacy. This review summarizes our current understanding of the participation of the ERK kinases in DDR.

## 1 INTRODUCTION

DDR guards genome integrity *via *sensing DNA lesions, activating checkpoints to halt cell cycle progression, and repairing DNA damage [1]. This process is initiated and coordinated by two apical kinases, ataxia-telangiectasia mutated (ATM) and ATR (ATM- and Rad3-related). ATM and ATR subsequently upregulate p21^CIP1^, an inhibitor of cyclin-dependent kinases (Cdks) [2, 3] and activate checkpoint kinases 1/2 (CHK1/2). CHK1/2 then inactivates Cdc25C which is required for the activation of Cdk1 and Cdk2 [4]. Collectively, the activation of ATM and ATR initiates checkpoints to prevent cell cycle progression [1].

Consistent with ERK kinases being a major player in promoting cell cycle progression [5], ERK1 and ERK2 also contribute to DDR [6]. A variety of genotoxic agents have been shown to activate the ERK kinases and ERK activity modulates DDR [7]. Consistent with the apical roles of ATM and ATR in DDR, DNA damage-induced ERK activation has been reported to be a downstream event of ATM and ATR [8, 9]. On the other hand, ERK activation also facilitates the activation of ATM and ATR [10, 11]. The impact of the MEK-ERK pathway on checkpoint activation in DDR is cell type dependent [6]. While ERK activity facilitates DNA damage-induced cell cycle arrest and apoptosis in a several mammalian cell lines and Drosophila [12, 7, 13, 9, 14, 15], ERK activation also prevents DNA damage-induced apoptosis in myeloma and leukemia [16, 17]. In this review, we will briefly discuss the ERK system, ATM/ATR-initiated DDR, and experimental evidence showing the interplay between ERK and DDR. Finally, we will briefly discuss strategies of using MEK inhibitors in cancer therapy.

## 2 THE CORE FEATURES OF ATM AND ATR ACTIVATION DURING DDR

DNA damage response is the critical surveillance mechanism that maintains genome integrity and ensures the accurate transmission of genetic information between generations in eukaryotic cells. DDR is mediated by three apical PI3 kinase related kinases (PIKKs), ATM, ATR, and DNA-PKcs (CS: catalytic subunit) [1]. PIKKs possess typical structural features, including the FRAP-ATM-TRRAP (FAT), kinase, and C-terminal FAT (FATC) domain [18, 19]. In line with these structural features, activation of PIKKs share common features, including the interactions with specific proteins and the association with unique DNA lesions [20]. 

ATM activation is induced by double stranded DNA breaks (DSBs). In human cells, ATM exists as an inactive dimer, which is converted into active monomers in response to DSBs *via *autophosphorylation at serine 1981 (S1981) [21]. This autophosphorylation stabilizes the association of ATM with DSBs [22]. Accumulating evidence demonstrates that the MRE11-RAD50-NBS1 (MRN) complex plays a pivotal role in ATM signalling. Patients with Nijmegen Breakage Syndrome (NBS) or Ataxia-Telangiectasia-like Disorder (ATLK) display similar clinical and cellular phenotypes with AT patients, including microcephaly, immunodeficiency, being prone to the development of malignancy, sensitization to ionizing radiation (IR), genomic instability and checkpoint defects following DNA damage. These three diseases are caused by mutations in NBS1, MRE11, and ATM, respectively [23]. These observations, therefore, provide strong genetic and cellular evidence showing that the MRN complex and ATM are involved in the same DDR pathway. Biochemically, the MRN complex directly interacts with ATM, resulting in ATM being recruited to DSBs [24, 25, 26], and converting ATM from inactive dimers to active monomers [24, 27]. This association of ATM and the MRN complex is mediated by a direct interaction between ATM and NBS1 [28]. The association with the MRN complex at DSBs activates ATM [27] and ATM subsequently phosphorylates numerous targets to coordinate checkpoint activation and DNA damage repair [1].

**Fig. (1):**
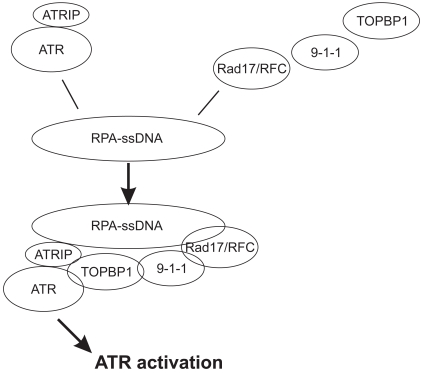
An illustration of ATR activation. 1) The ATR-ATRIP complex binds to RPA-ssDNA; 2) Rad17/RFC, 9-1-1, and TOPBP1 are sequentially loaded onto RPA-ssDNA; 3) TOPBP1 interacts with the ATR-ATRIP complex, resulting in ATR activation.

While ATR is activated by multiple types of DNA lesions, including DSBs, base adducts, crosslinks, and replication stress [18]; its activation is primarily mediated by the structure containing single-stranded DNA (ssDNA) [29, 30]. Association of ATR with replication protein A (RPA)-coated ssDNA is required for ATR activation. ATR binds directly to the ATR interacting protein (ATRIP). This association does not depend on DNA damage but is required for the stabilization of both ATR and ATRIP, as knockdown of one causes the reduction of the other [31]. ATR function depends on ATRIP in human cells and its homologues in yeast, Rad26 in *Schizosaccharomyces prombe* and DDC2/LCD1/PIE1 in *Saccharomyces cerevisiae*. Loss of ATRIP in yeast and human cells recapitulate the same phenotypes as loss of ATR [31, 32, 33, 34, 35, 36]. ATRIP binds to RPA-coated ssDNA and thereby results in the recruitment of ATR to the DNA lesion [30, 37, 38]. Although the association of the ATR-ATRIP complex with RPA-coated ssDNA is required, it is insufficient to increase ATR-derived kinase activity. Biochemical analysis revealed that TOPBP1 (topoisomerase-binding protein 1), the mammalian homologue of yeast Cut5/Dbp11, significantly enhanced the kinase activity of the ATR-ATRIP complex *in vitro* [39]. In line with the RPA-coated ssDNA being the primary structure leading to ATR activation; TOPBP1 is recruited to RPA-coated ssDNA independent of the ATR-ATRIP complex, and requires the Rad17/RFC (replication factor C) and the Rad9-Rad1-Hus1 (9-1-1) complex. Rad17/RFC binds to RPA-ssDNA (Fig. **1**) [40, 20], which loads the 9-1-1 complex [41, 42] and subsequently recruits TOPBP1 [43, 44]. This recruitment allows TOPBP1 to activate ATR *via *binding to both ATR and ATRIP subunits (Fig. **1**) [39, 45]. ATR activation coordinates a global cellular response to prevent cell cycle progression into mitotic phase (S phase arrest or induction of apoptosis) and to initiate DNA damage repair [18]. ATR accomplishes these multiple functions by phosphorylation of a variety of down-stream targets, including the serine 139 (S139) in the SQE motif located on the tail of histone H2AX (γH2AX), the S15 of p53, and the S345 of CHK1 [1, 46, 47]. 

## 3 ERK KINASES PLAY A ROLE IN DDR

It is well recognized that DDR is intimately linked to cell division. One of the major tasks of ATM and ATR activation is to initiate checkpoint controls to halt cell cycle progression in order to coordinate the repair of DNA lesions [1]. Activation of checkpoints leads to the up-regulation of p21^CIP1^ and the inactivation of CDC25C *via *activation of CHK1 and CHK2 [48, 1]. These events collectively inhibit the kinase activity of Cdk1 and Cdk2, leading to cell cycle arrest. Therefore, inhibition of Cdk activity or arrest of cell division is a downstream event of ATM and ATR. Recent developments, however, demonstrated that cell division contributes more to DDR than only being a downstream event. Cdk activity promotes the proper activation of ATM and ATR in response to DNA damage [49, 50]. This may explain, at least in part, a long-term observation that malignant cells are more sensitive to genotoxic agents than cells in normal tissues [51]. Direct evidence supporting this concept was the recent demonstration that directly blocking cell division *via *loss of CDC25 protected intestinal mucosa from damage induced by irinotecan, a commonly used chemotherapeutic/genotoxic agent in treating patients with colon cancer [52]. Consistent with the theme that the mechanism regulating cell proliferation actively engages DDR, ERK1 and ERK2 kinases, also contributes to DDR *via *mechanisms that are dependent or independent on p53 as well as lying downstream of ATM and ATR or facilitates the activation of ATM and ATR.

### 3.1 ERK MAP Kinases

ERK1 and ERK2 (ERK1/2) kinases belong to the mitogen-activated protein kinase (MAPK) family and are activated by two highly conserved upstream kinases, MEK1/2 (MAPK kinase). In addition, MEK1/2 kinases are activated by Raf (MAPK kinase kinase) [53, 54]. MEK1/2 activates ERK1/2 kinases through phoshorylation of threonine 183 (Thr183) and tyrosine 185 (Tyr185) at the Thr-Glu-Tyr site [54]. In addition to the Raf and MEK upstream kinases, ERK activation is facilitated by scaffolding proteins. One such protein is the kinase suppressor of Ras 1 (KSR1) which binds to MEK and thereby facilitates ERK activation [53, 55, 56]. ERK functions in multiple signalling pathways *via *the phosphorylation of numerous target proteins involved in cell proliferation, differentiation, and apoptosis [53, 57, 58]. In addition, ERK1 and ERK2 achieve substrate specificity using two mechanisms. ERK1 and ERK2 phosphorylates serine/threonine (S/T) residues that are immediately followed by a proline (P) (S/T-P motif) [59, 60]. Many ERK substrates contain one or two docking sites to facilitate their association with ERK, thereby enhancing their phosphorylation [53, 61]. The consensus docking sites for DEJL (docking site for ERK and JNK, LXL) and DEF (docking site for ERK, FXFP) are (R/K)_2_X_2-6_LXL (X: any residue) [61] and FXFP [62, 53], respectively. 

### 3.2 ERK Modulates DDR in a Cell-Dependent Manner

Activation of ERK1/2 is commonly observed in multiple cell lines by a variety of genotoxic agents. Etoposide (ETOP), adriamycin, UV, ionizing radiation (IR), hyroxyurea (HU), and mitomycin C (MMC) were shown to activate ERK1/2 in mouse embryonic fibroblast (MEFs), NIH3T3, MCF7, H9c2, U87, keratinocytes cells, and normal human diploid fibroblasts (IMR90 and 501T) [7, 13, 63, 64, 9, 8, 65, 66]. Both ETOP and IR induced ERK activation reaching a plateau at one hour in MEFs [7, 63]. However, cisplatin activated ERK in HeLa, A2780, and HEI-OCI auditory cells [12, 67, 68], which was observed at 15 minutes and reached a peak at 30 minutes [68]. Activation of ERK was also observed in T98G cells in response to cisplatin and UV [69]. In addition, BRCA1, which induces the activation of CHK1 and WEE1, also activated ERK [70]. Collectively, the kinetics of ERK activation induced by a variety of DNA damage reagents are regulated by cellular contents during DDR.

ERK activity also modulates checkpoint activation in a cellular context-dependent manner. Inhibition of ERK activation with the commonly used MEK inhibitors (PD98059 and U0126) and a dominant negative MEK1K97M attenuates ETOP and HU-induced G2/M and S phase arrest in several cell lines, including NIH3T3, MCF7, MEF, and HCT116 [7, 13, 9]. Consistent with these observations, knockdown of either ERK1 or ERK2 compromises ETOP and HU-induced G2/M and S phase arrest in MCF7 cells [10, 11]. Conversely, enforced activation of ERK1/2 using a constitutively active MEK1Q56P sensitizes S phase checkpoint in response to HU [13]. The MEK-ERK pathway constitutes an intrinsic component of IR-initiated checkpoint in Drosophila [15]. In podocytes, Cyb-9 induces DNA damage, cell cycle arrest, and ERK activation and inhibition of ERK activation reduces cell cycle arrest [71]. MMC activates ERK in neurons, which promotes MMC-induced apoptosis [14]. Consistent with these observations, ERK activity also contributes to ETOP-induced apoptosis in NIH3T3 cells [7], cisplatin-induced apoptosis in HeLa cells [12, 72], as well as cisplantin- and UV-induced apoptosis in human glioblastoma T98G cells [69]. On the other hand, ERK activation was reported to inhibit DNA damage-induced apoptosis in myeloma and leukemia cells in response to the Chk1 inhibitor UCN-01 and cytarabien (AraC), respectively [16, 17]. In human multiple myeloma (MM) cells, an inhibitor of CHK1 induces DNA damage, which is accompanied by ERK activation. Inhibition of ERK activation sensitizes UCN-01, a Chk1 inhibitor, and induced DNA damage and apoptosis in MM cells [16]. Similar observation was also reported in acute myelogenous leukemia (AML), NB4, and HL60 cells in response to cytarabien (AraC)-induced DNA damage [17]. 

Additionally, ERK activation also contributes to cell cycle reinitiation following DNA damage-induced cell cycle arrest. It was demonstrated in KSR1 deficient mouse embryonic fibroblasts (MEF), IR, UV, and MMC-induced ERK activation were inhibited and that re-expression of KSR1 in KSR1^-/-^ MEFs rescued the defects of ERK activation in response to IR, UV, and MMC [63]. Detailed analysis of MMC-induced DDR in KSR1^-/-^ MEFs revealed that although MMC induced KSR1^-/-^ MEFs to undergo G2/M arrest, the cells were unable to recover from the arrest. Complementation of KSR1^-/-^ MEFs with wild type KSR1 but not a KSR1 mutant that is incapable of binding to ERK enabled the cells to recover from MMC-induced G2/M arrest [63]. Collectively, these observations demonstrate that ERK activation plays a critical role in the re-entry of cell cycle following MMC-induced DNA damage [63]. Furthermore, ERK activation mediates adriamycin and ETOP-induced up-regulation of glucose transporter 3 in HeLa cells [73] as well as playing a role in IR-induced activation of NF-κB [74]. Taken together, a large body of evidence reveals that ERK kinases play an important role in DDR.

While the underlying mechanisms responsible for DNA damage-induced ERK activation remain elusive, the accumulating evidence indicates that MEK mediates ERK activation in DDR. The inhibition of MEK activation with MEK inhibitors (PD98059, U0126), a dominant negative MEK1K97M, and MEK siRNA inhibited ERK activation that is induced by a variety of genotoxic agents [7, 13, 9, 8, 64, 16, 10, 11]. However, whether DNA damage activates MEK *via *Raf remains to be demonstrated. 

While MMC activates ERK in wild type but not p53 deficient MEFs [66], ETOP induces ERK activation in wild type and p53^-/-^ MEFs [7]. Additionally, both cisplatin and UV robustly activate ERK in human glioblastoma T98G cells lacking functional p53 [69]. Therefore, while p53 may contributes to DNA damage-induced ERK activation under certain conditions, p53 function may not be required. This will be in line with the observation that at least half of human cancers express mutant p53. Consistent with ATM being the apical kinase in IR-initiated DDR, it has been shown that IR-induced ERK activation in U87 cells is partially regulated by ATM [8]. Similar observations were also obtained from photolysis-induced DSBs [75].

### 3.3 ERK Kinases Facilitate the Activation of ATM and ATR

Recent developments have advanced our understanding of DNA damage-initiated activation of PIKKs. It is becoming clear that the activation of PIKKs is mediated by the association with specific DNA structures and proteins, like NBS1 for ATM and TOPBP1 for ATR [76, 45, 20]. However, the detailed mechanism responsible for ATM and ATR activation in DDR remain largely elusive. It is thus intriguing that DNA damage-induced ERK activation contributes to the activation of ATM and ATR. 

Cisplatin was reported to robustly activate ERK in human ovarian carcinoma A2780 cells. Inhibition of ERK activation using PD98059 reduced the phosphorylation of p53 at S15 in response to cisplatin [67]. In addition, inhibition of ERK activation with U0126 reduces doxorubicin-induced p53 S15 phosphorylation in H9c2 cells [64]. As S15 is not followed by a proline (P), it is thus very unlikely that ERK1/2 can directly phosphorylate p53 S15. The S15 is followed by QE (15-SQE-17) (http://www.uniprot.org/uniprot/P04637). Consistent with the S/T-QE sequence is the well demonstrated phosphorylation site for ATM and ATR [1], p53 S15 is phosphorylated by ATM/ATR in DDR [1]. It is, thus, likely that ERK enhances p53 S15 phosphorylation, as reported by Persons *et al*. [67] and Liu *et al*. [64], *via *facilitation of the activation of ATM and or ATR. This possibility is supported by the observation that IR-induced nuclear foci of S1981 phosphorylated ATM was significantly reduced in U87 cells when ERK activation was inhibited by PD184352, a MEK inhibitor [8]. A similar observation was also reported in MCF7 cells. U0126 (one of most commonly used MEK inhibitors) compromises IR-induced ATR activation as well as the downstream events of ATR, including CHK1 activation, CDC25 inactivation, and CDC2 inactivation [9]. Consistent with ERK1/2 being the major, if not the sole targets of MEK [53], it was recently demonstrated that knockdown of either ERK1 or ERK2 significantly reduced ATM activation (ATM S1981 phosphorylation and the nuclear foci of S1981 phosphorylated ATM) in response to ETOP, and thereby attenuated phosphorylation of the ATM substrates, including the S139 of H2AX (γH2AX), S15 of p53, and T68 of CHK2. As CHK2 inactivates CDC25C *via *phosphorylation of CDC25C S216, resulting in G2/M arrest [46, 3, 4], knockdown of either ERK1 or ERK2 reduced ETOP-induced CDC25C S216 phosphorylation and significantly compromised ETOP-induced G2/M arrest in MCF7 cells [10].

Hydroxyurea (HU) induces stalled replication forks, which primarily activates ATR to initiate the S-phase checkpoints [77]. HU activated ERK kinase in MCF7 cells, which facilitated ATR activation [13, 11]. Additionally, inhibition of ERK activation by using MEK inhibitors (PD98059 and U0126) and a dominant negative MEK1K97M reduced ATR nuclear foci, phosphorylation of ATR targets [p53 S15 and H2AX S139 (γH2AX)], S phase arrest in response to HU in NIH3T3, MCF7, MEF, and HCT116 cells [13]. The inhibition of ERK activation also attenuated CDC2 tyrosine (Y) 15 phosphorylation induced by HU [13, 11]. Phosphorylation of Y15 inactivates CDC2, whose activity is required for mitotic entry [78]. Therefore, inhibition of ERK activation may reduce HU-induced S phase arrest by improperly facilitating CDC2 activation. Conversely, ectopic expression of a constitutively active MEK1Q56P sensitized HU-induced formation of the nuclear foci of ATR and γH2AX [13]. Furthermore, knockdown of either ERK1 or ERK2 reduced HU-induced ATR activation, CHK1 S345 phosphorylation, p53 S15 phosphorylation, and γH2AX [11]. This was associated with compromising HU-induced S phase arrest and CDC2 Y15 phosporylation [11]. Intriguingly, it was observed that knockdown of either ERK1 or ERK2 resulted in a significant accumulation of ATR in the nucleolus when cells were treated with HU. DNA damage was reported to induce protein trafficking in to and out of the nucleolus, thereby regulating DDR [79]. In general, proteins with functions in promoting DDR, including p14ARF [80], WRN (Werner Syndrome Protein) [81, 82], PARP-1 (Poly(ADP-ribose) polymerase-1) [83], and BRCA1 [84], translocate from the nucleoli to nucleoplasm upon DNA damage. However, proteins that inhibit DDR, like MDM2, relocate from the nucleoplasm into the nucleolus [85]. Therefore, the net result of these protein traffics is to promote DDR. It is thus plausible that the observed relocation of ATR to the nucleolus in HU-treated ERK1 or ERK2 knockdown cells may sequester ATR in the nucleoli. This would be consistent with the observation that the reduction of either ERK1 or ERK2 has no effect on HU-induced recruitment of RPA60 to ssDNA, indicating that ERK kinases may not play a major role in the formation of RPA-coated ssDNA [11]. Taken together, accumulating evidence demonstrates that ERK1/2 facilitates DNA damage-induced ATR activation.

## 4 FUTURE PERSPECTIVES IN ERK-FACILITATED DDR

**Fig. (2):**
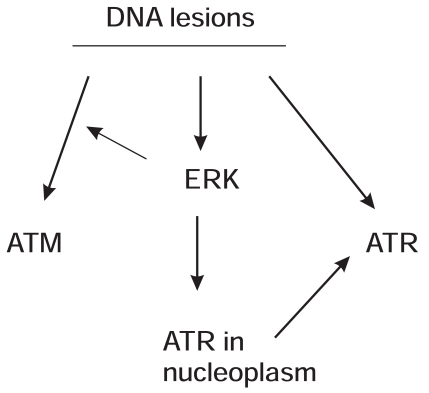
A model illustrating ERK-facilitated activation of ATM and ATR during DDR. 1) DNA lesions lead to activation of ATM, ATR, and ERK; 2) ERK facilitates ATM activation; 3) ERK executes the proper ATR activation at least in part by ensuring ATR stay in the nucleoplasm.

**Fig. (3):**
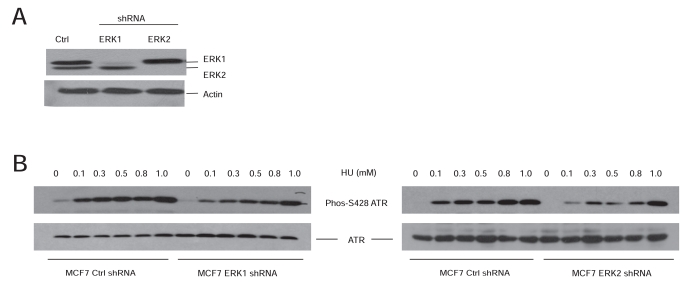
ERK facilitates ATR S428 phosphorylation in response to HU. **A**) MCF7 cells were stably infected with empty vector (Ctrl), ERK1 shRNA, and ERK2 shRNA. The expression of ERK1, ERK2, and actin was examined by western blot using the specific antibodies. **B**) MCF7 Ctrl (control), ERK1 shRNA and ERK2 shRNA cells were treated with HU at the indicated doses for 24 hours, followed by analysis of ATR S428 phosphorylation (Phos-S428 ATR) (Cell Signaling, 1:1000) and total ATR (Calbiochem, 1:1000).

Research carried out in the last decade gradually consolidated the contributions of the MEK-ERK kinases to DDR. While being heavily investigated for a long period of time, the underlying mechanisms whereby DNA damage activates ATM and ATR, two apical kinases in DDR, remain unclear. The ERK kinases may add to the missing pieces regarding how DNA lesions activate ATM and ATR. ERK kinases facilitate the activation of apical ATM or ATR kinases in response to a variety of DNA damages (Fig. **2**). In the case of ATR, ERK kinases facilitate ATR activation at least in part by preventing the accumulation of ATR in the nucleolus (Fig. **2**). While the observations that MEK-ERK kinases facilitate the proper activation of ATM and ATR do add to the importance of ERK in DDR regulation, the underlying mechanisms remain to be determined. Will ERK directly phosphorylate ATM/ATR or indirectly phosphorylate other components that are involved in ATM/ATR activation? Will these phosphorylations play major roles in ATM/ATR activation? The ATM protein contains several potential ERK phosphorylation sites (S/T-P) and 2 potential ERK docking sites, DEJL domains (1150-RKSVLLTL-1157 and 2302-KKEQSLAL-2309). These sites match to the consensus sequence for the DEJL domain (R/K)_2_X_2-6_LXL [61]. The phosphorylation of ATR at S428 was reported in DDR [86]. We have shown that HU robustly induces ATR S428 phosphorylation and that knockdown of ERK1 or ERK2 dramatically reduces this event (Fig. **3**). The kinases that phosphorylate S428 and the impact of this event on ATR function remain unknown. However, S428 is followed by a proline (P) (DGISPKRRR), a site that matches the substrate specificity of ERK kinases [59]. Additionally, human ATR contains a candidate DEF motif, 983-FDFP-985. This site matches the consensus of the DEF ERK docking site FXFP [62, 53]. It is thus a possibility that ERK may phosphorylate ATR at S428.

## 5 OPTIMIZATION OF MEK INHIBITORS INVOLVED IN CANCER THERAPY

Ras and Raf [54], ERK kinases are commonly activated abnormally in most human cancers due to the converging site in transmitting signals derived from growth factors. The amplification of upstream growth factor receptors takes place frequently in cancer, including the epidermal growth factor receptor (EGFR) in solid tumors [87] and BCR-ABL [fusion of the *Abl1* oncogene gene on chromosome 9 to the BCR (breakpoint cluster region) gene on chromosome 22] in chronic myeloid leukemia (CML) [88]. Additionally, the amplification of the *ras* oncogene is detected in approximately 30% of human cancers [89]. Mutations leading to the activation of BRAF (the B isoform of RAF) were detected in 27-70% of melanoma, 36-53% of papillary thyroid cancer, 5-22% of colorectal cancer, and 30% of ovarian cancer [90]. In line with abnormal activation of the ERK kinases being one of the common events in human cancers, ERK kinases are well regarded to drive cancerous cell proliferation and promote other oncogenic events, including survival and angiogenesis [91, 92]. Therefore, inhibition of MEK-mediated ERK activation may be an effective option in cancer therapy. Indeed, several highly specific MEK inhibitors have been developed, including PD184352/CI-1040 (Pfizer), PD0325901 (Pfizer), AZD6244 (ARRY-142886 or Selumetinib) (Astra Zeneca) and RDEA119 (Ardea Biosciences) [93]. While these small molecule MEK inhibitors are highly specific and effective in preclinical settings, they are, however, not effective in clinical trials on a variety of tumors. PD184352, the first MEK inhibitor entering clinical trials, failed to show encouraging results when treating patients with advanced non-small cell lung, breast, colon, and pancreatic cancer [94]. PD0325901 also did not produce overwhelming positive outcomes in clinical trials on patients with breast, colon, melanoma, and non-small cell lung cancer (NSCLC) [95, 96]. This was also the situation for a newly developed MEK inhibitor AZD6244 when examined in clinical trials on melanoma and NSCLC [97, 98]. While better designed clinical trials on selected patients with tumors that are dictated to ERK activation caused by BRAF or KRAS activation [99, 100], might have yielded more positive outcomes, it is uncertain how the potential positive results might be. This is because 1) in clinical trials on melanoma, only 12% of tumors with BRAF mutations were partially responsive to AZD6244 [97], 2) NSCLCs with KRAS mutations display heterozygous responses to MEK inhibitors, and 3) a minor proportion (21%) of patients having BRAF V600 mutation showed responses to the MEK inhibitor GSK1120212 [101, 102]. Taken together, clinical trials using a variety of MEK inhibitors were unable to produce outcomes that are proportional to the prevalence of ERK activation in human cancers.

Although there are complex factors that are certainly contributing to the lack of success for MEK inhibitors, such as the design of clinical trials, limitation of tolerable doses being used, and the development of resistance. The role of ERK in tumorigenesis may also be a contributing factor. ERK activity is widely regarded to provide proliferation signals to cancerous cells, the main underlying reason to target ERK activation by using MEK inhibitors. However, recent developments have clearly demonstrated that ERK kinases play an important role in DNA damage response (DDR). This is consistent with the observation that activation of the RAF-MEK-ERK pathway is commonly associated with chemotherapy and radiotherapy [103] as chemotherapeutic drugs commonly induce DNA damage [104]. Therefore, applications involving MEK inhibitors in cancer therapy should be considered very carefully as maintaining genome integrity is a driving force of tumor suppression. 

The contribution of ERK to DDR outlines a scientific background for a combinational therapy involving genotoxic drugs and MEK inhibitors. As DNA damage-induced ERK activation inhibited DDR-associated apoptosis in myeloma and leukemia [16, 17], inhibition of ERK activation will be expected to enhance the efficacy of genotoxic drugs on these cancers. However, for tumors not associated with the hematopoietic system, ERK activation sensitizes DNA damage-induced checkpoint activation [7, 9-15, 69, 71, 72]. Therefore, inhibition of ERK activation in these cancers may also enhance the genotoxic effect of chemotherapeutic drugs. This may be caused by the accumulation of DNA lesions due to impaired checkpoint activation when ERK activation is inhibited. This concept is supported by a recent report showing that the MEK inhibitor ADZ6244 enhanced radiation-induced reduction of A549-derived xenograft tumors [105]. However, inhibition of ERK may compromise checkpoint activation and thereby allowing cells to proliferate in the presence of DNA lesions. This may lead to accumulation of mutations and thus contribute to cancer progression, which might be attributable to the resistance or the inefficiency of MEK inhibitors in cancer therapy. Therefore, a key factor in determining a regime of MEK inhibitors and genotoxic drugs in treating solid cancers is whether this combinational therapy will lead to a catastrophic result due to inefficient repair of DNA lesions or continuous cell proliferation in the presence of DNA lesions.
